# Postpartum haemorrhage in anaemic women: assessing outcome measures for clinical trials

**DOI:** 10.1186/s13063-022-06140-z

**Published:** 2022-03-18

**Authors:** Amy Brenner, Ian Roberts, Eni Balogun, Folasade Adenike Bello, Rizwana Chaudhri, Charlotte Fleming, Kiran Javaid, Aasia Kayani, Mwansa Ketty Lubeya, Raoul Mansukhani, Oladapo Olayemi, Danielle Prowse, Bellington Vwalika, Haleema Shakur-Still

**Affiliations:** 1 London School of Hygiene and Tropical Medicine, Clinical Trials Unit, Keppel Street, London, WC1E 7HT UK; 2 Department of Obstetrics and Gynaecology, University College Hospital, Ibadan, Oyo State Nigeria; 3 Global Institute of Human Development, Shifa Tameer-e-Millat University, Islamabad, 44000 Pakistan; 4 Women and Newborn Hospital, University Teaching Hospital, Department of Obstetrics and Gynaecology, Nationalist Road, Private Bag RW1X, 10101 Lusaka, Zambia

**Keywords:** Anaemia, Bleeding, Haemoglobin, Outcome measure, Postpartum haemorrhage, Randomised controlled trial, Tranexamic acid, Treatment effect, WOMAN-2 trial

## Abstract

**Background:**

Postpartum haemorrhage (PPH) is a leading cause of maternal mortality worldwide. Maternal anaemia greatly increases the risk of PPH, and over a third of all pregnant women are anaemic. Because anaemia reduces the oxygen-carrying capacity of the blood, anaemic women cannot tolerate the same volume of blood loss as healthy women. Yet the same blood loss threshold is used to define PPH in all women. The lack of an established PPH definition in anaemic women means the most appropriate outcome measures for use in clinical trials are open to question. We used data from the WOMAN-2 trial to examine different definitions of PPH in anaemic women and consider their appropriateness as clinical trial outcome measures.

**Main body:**

The WOMAN-2 trial is assessing tranexamic acid (TXA) for PPH prevention in women with moderate or severe anaemia at baseline. To obtain an accurate, precise estimate of the treatment effect, outcome measures should be highly specific and reasonably sensitive. Some outcome misclassification is inevitable. Low sensitivity reduces precision, but low specificity biases the effect estimate towards the null. Outcomes should also be related to how patients feel, function, or survive. The primary outcome in the WOMAN-2 trial, a ‘clinical diagnosis of PPH’, is defined as estimated blood loss > 500 ml or any blood loss within 24 h sufficient to compromise haemodynamic stability. To explore the utility of several PPH outcome measures, we analysed blinded data from 4521 participants. For each outcome, we assessed its: (1) frequency, (2) specificity for significant bleeding defined as shock index ≥1.0 and (3) association with fatigue (modified fatigue symptom inventory [MFSI]), physical endurance (six-minute walk test) and breathlessness. A clinical diagnosis of PPH was sufficiently frequent (7%), highly specific for clinical signs of early shock (95% specificity for shock index ≥1) and associated with worse maternal functioning after childbirth.

**Conclusion:**

Outcome measures in clinical trials of interventions for PPH prevention should facilitate valid and precise estimation of the treatment effect and be important to women. A clinical diagnosis of PPH appears to meet these criteria, making it an appropriate primary outcome for the WOMAN-2 trial.

**Trial registration:**

ClinicalTrials.gov NCT03475342, registered on 23 March 2018; ISRCTN62396133, registered on 7 December 2017; Pan African Clinical Trial Registry PACTR201909735842379, registered on 18 September 2019.

## Background

Postpartum haemorrhage (PPH) is a leading cause of maternal mortality worldwide, responsible for over 70,000 deaths annually [1]. Maternal anaemia greatly increases the risk of PPH [2, 3]. Over a third of all pregnant women (around 30 million) are anaemic, with a high prevalence in sub-Saharan Africa and South Asia [4]. Because anaemia reduces the oxygen-carrying capacity of the blood, anaemic women are more vulnerable to tissue hypoxia, morbidity and death after PPH [5, 6].

Primary PPH is usually defined as blood loss ≥500 ml from the genital tract within 24 h of a vaginal birth [7]. This definition, proposed by a WHO working group in 1989, uses the same threshold for all women. Despite recognising the need for an alternative definition in anaemic women, no specific criteria were proposed [8]. The core outcome set for PPH prevention trials does not consider anaemia [9].

Given the lack of an established definition of PPH in anaemic women, the most appropriate outcome measures for use in clinical trials are open to question. We used data from the WOMAN-2 trial to examine different definitions of PPH in anaemic women and consider their appropriateness as clinical trial outcome measures.

### Criteria to assess PPH outcome measures

The WOMAN-2 trial is examining tranexamic acid (TXA) for PPH prevention in women with moderate (Hb 70–99 g/L, *n* = 3714, 82%) or severe (Hb < 70 g/L, *n* = 805, 18%) anaemia at baseline. Women are randomly allocated to receive 1 g of TXA or matching placebo as soon as possible after cord clamping. The primary outcome, a ‘clinical diagnosis of PPH’, may be defined as estimated blood loss > 500 ml or any blood loss within 24 h sufficient to compromise haemodynamic stability. Haemodynamic instability is based on clinical judgement and assessed using clinical signs (low systolic blood pressure, tachycardia, reduced urine output) that require an intervention (e.g. intravenous fluids)) [10].

In a clinical trial, the primary outcome should facilitate valid and precise estimation of the treatment effect and be related to how patients feel, function or survive [11, 12]. Some outcome misclassification is inevitable. Table 1 shows the potential impact of sensitivity and specificity on the relative risk (RR) in the WOMAN-2 trial. Assuming 6% of the placebo group have a PPH and TXA reduces this risk by 25% (RR = 0.75), a sample size of 10,000 should provide 90% power [10]. Low sensitivity (many false negatives) reduces precision but the RR remains the same, whereas low specificity (many false positives) biases the RR towards the null [13].

**Table 1:** Impact of sensitivity and specificity on the treatment effect estimate in a randomised trial. Hypothetical example based on the WOMAN-2 trial of 10,000 women (5000 per arm), assuming a true placebo group event rate of 6% and a true relative risk of 0.75

Varying specificity, 100% sensitivity				Varying sensitivity, 100% specificity			
Specificity	Outcome events (***n***)		RR (95% CI)	Sensitivity	Outcome events (***n***)		RR (95% CI)
	TXA	Placebo			TXA	Placebo	
100%	225	300	0.75 (0.62−0.88)	100%	225	300	0.75 (0.62−0.88)
95%	464	535	0.87 (0.78−0.96)	95%	214	285	0.75 (0.61−0.89)
90%	703	770	0.91 (0.84−0.98)	90%	203	270	0.75 (0.61−0.89)
85%	941	1005	0.94 (0.88−1.00)	85%	191	255	0.75 (0.60−0.90)
80%	1180	1240	0.95 (0.90−1.00)	80%	180	240	0.75 (0.59−0.91)

*RR* relative risk, *CI* confidence interval, *TXA* tranexamic acid

To explore the utility of several PPH outcome measures, we analysed blinded data from 4521 participants recruited to 14th July 2021. For each outcome, we assessed its: (1) frequency, (2) specificity for significant bleeding and (3) importance to women. To assess frequency, we considered the sample size calculation for the trial—for 90% power to detect a 25% reduction in PPH with TXA, a minimum event rate of 6% in the placebo group is required, with an event rate of 4.5% in the TXA group and therefore 5.25% overall. To assess specificity, we used a shock index (postpartum heart rate/systolic blood pressure) ≥1.0 as the ‘gold standard’ for the cardiovascular impact of bleeding (see Table 2) [14–17]. To assess importance to women, we examined each outcome’s association with fatigue (modified fatigue symptom inventory [MFSI]), physical endurance (6-min walk test) and breathlessness (patient-reported outcome post-walk test), (see Table 3).

**Table 2:** Cumulative incidence of PPH outcome measures and their diagnostic accuracy for early shock defined as shock index ≥1

PPH definition	SI ≥ 1		SI < 1		Total		Sensitivity	Specificity
	*n*	(%)	*n*	(%)	*N*	(%)		
**Clinical diagnosis of PPH**								
Yes	109	(31)	208	(5)	317	(7)	31%	95%
No	240	(69)	3956	(95)	4196	(93)		
Total	349	(100)	4164	(100)	4513	(100)		
**Estimated blood loss ≥ 500 ml**								
Yes	95	(27)	274	(7)	369	(8)	27%	93%
No	254	(73)	3890	(93)	4144	(92)		
Total	349	(100)	4164	(100)	4513	(100)		
**Total blood volume lost ≥ 15%**								
Yes	48	(14)	69	(2)	117	(3)	14%	98%
No	301	(86)	4095	(98)	4396	(97)		
Total	349	(100)	4164	(100)	4513	(100)		
**Peripartum Hb drop ≥ 20 g/L**^**a**^								
Yes	47	(14)	111	(3)	158	(4)	14%	97%
No	297	(86)	3990	(97)	4287	(96)		
Total	344	(100)	4101	(100)	4445	(100)		
**Peripartum Hb drop ≥ 10%**^**a**^								
Yes	105	(31)	604	(15)	709	(16)	31%	85%
No	239	(69)	3497	(85)	3736	(84)		
Total	344	(100)	4101	(100)	4445	(100)		
**Calculated blood loss ≥ 1000 ml**^**a**^								
Yes	70	(20)	298	(7)	368	(8)	20%	93%
No	274	(80)	3799	(93)	4073	(92)		
Total	344	(100)	4097	(100)	4441	(100)		
**RBC transfusion within 24 h after delivery**								
Yes	119	(34)	1010	(24)	1129	(25)	34%	76%
No	226	(66)	3121	(76)	3347	(75)		
Total	345	(100)	4131	(100)	4476	(100)		
**IV fluid within 24 h after delivery**								
Yes	200	(58)	1716	(42)	1916	(43)	59%	58%
No	139	(40)	2331	(56)	2470	(55)		
Total	339	(98)	4047	(98)	4386	(98)		
**TXA within 24 h after delivery**								
Yes	87	(25)	169	(4)	256	(6)	25%	96%
No	262	(76)	3994	(97)	4256	(95)		
Total	349	(101)	4163	(101)	4512	(101)		
**Postpartum uterotonics**								
Yes	133	(39)	1174	(28)	1307	(29)	38%	72%
No	216	(63)	2990	(72)	3206	(72)		
Total	349	(101)	4164	(101)	4513	(101)		

*PPH* postpartum haemorrhage, *SI* shock index, *Hb* haemoglobin, *RBC* red blood cell, *IV* intravenous, *TXA* tranexamic acid

^a^Postpartum Hb corrected for RBC transfusions and IV fluids received between randomisation and postpartum Hb test

**Table 3:** Association of PPH with measures of maternal functioning after birth

PPH definition	Fatigue (MSFI score)			6-min walk test (metres)^**a**^			Moderate-extreme breathlessness			
	***N***	Mean ± SD	Dif. in means (95% CI)	***N***	Mean ± SD	Dif. in means (95% CI)	***n***	***N***	(%)	RR (95% CI)
**Clinical diagnosis of PPH**										
Yes	304	3.8 ± 20.3	8.0 (5.7–10.4)	291	154.1 ± 85.3	− 21.3 (− 31.6 to − 11.0)	46	281	(16)	1.97 (1.49–2.62)
No	4102	− 4.2 ± 15.5		3993	175.4 ± 97.7		327	3944	(8)	
**Blood loss ≥ 500 ml**										
Yes	353	2.0 ± 19.9	6.1 (4.0–8.3)	337	158.9 ± 85.5	− 16.7 (− 26.3 to − 7.0)	49	330	(15)	1.79 (1.35–2.37)
No	4052	− 4.1 ± 15.5		3946	175.3 ± 97.8		323	3894	(8)	
**Total blood volume lost ≥ 15%**										
Yes	111	4.4 ± 20.2	8.3 (4.4–12.1)	105	160.2 ± 87.7	− 14.2 (− 32.9 to 4.6)	16	102	(16)	1.82 (1.15–2.88)
No	4294	− 3.7 ± 15.8		4178	174.4 ± 97.2		356	3411	(10)	
**Peripartum Hb drop ≥ 20 g/L** ^**b**^										
Yes	150	4.7 ± 22.8	8.7 (5.0–12.4)	147	187.8 ± 104.5	13.8 (− 2.2 to 29.8)	27	141	(19)	2.31 (1.62–3.29)
No	4211	− 4.0 ± 15.5		4093	174.0 ± 97.0		335	4040	(8)	
**Peripartum Hb drop ≥ 10%** ^**b**^										
Yes	687	− 0.8 ± 19.7	3.5 (1.9–5.0)	670	180.9 ± 106.4	7.7 (− 1.0 to 16.3)	91	655	(14)	1.81 (1.45–2.26)
No	3674	− 4.3 ± 15.1		3570	173.2 ± 95.5		271	3255	(8)	
**Calculated blood loss ≥ 1000 ml** ^**b**^										
Yes	353	2.2 ± 21.5	6.4 (4.1–8.7)	347	184.1 ± 108.1	10.6 (− 1.3 to 22.4)	51	337	(15)	1.87 (1.42–2.46)
No	4004	− 4.3 ± 15.2		3889	173.6 ± 96.3		311	3840	(8)	
**RBC transfusion within 24 h after delivery**										
Yes	1105	0.4 ± 17.7	5.5 (4.4–6.7)	1046	151.1 ± 88.4	− 30.6 (− 36.9 to − 24.2)	105	1032	(10)	1.20 (0.97–1.49)
No	3267	− 5.1 ± 15.0		3205	181.6 ± 98.6		267	3162	(8)	
**IV fluid within 24 h after delivery**										
Yes	1863	− 0.6 ± 17.3	5.5 (4.5–6.4)	1818	161.0 ± 90.1	− 24.0 (− 29.8 to − 18.2)	211	1785	(12)	1.82 (1.49–2.22)
No	2422	− 6.1 ± 14.4		2349	185.0 ± 100.3		151	2326	(6)	
**TXA within 24 h after delivery**										
Yes	245	4.8 ± 20.9	9.0 (6.3–11.7)	240	148.9 ± 83.5	− 26.5 (− 37.5 to − 15.5)	38	230	(17)	1.97 (1.45–2.68)
No	4160	− 4.2 ± 15.5		4043	175.4 ± 97.6		335	3994	(8)	
**Postpartum uterotonics**										
Yes	1274	− 0.5 ± 17.8	4.5 (3.4–5.6)	1157	144.6 ± 92.4	− 40.2 (− 46.7 to − 33.8)	117	1118	(10)	1.27 (1.03–1.56)
No	3132	− 5.0 ± 15.0		3127	184.9 ± 96.4		256	3107	(8)	
**Shock index ≥ 1**										
Yes	331	0.3 ± 19.9	4.3 (2.1 to 6.5)	334	191.7 ± 103.0	19.3 (7.8 to 30.7)	44	325	(14)	1.60 (1.20 to 2.15)
No	4075	− 4.0 ± 15.6		3950	172.5 ± 96.3		329	3900	(8)	

*PPH* postpartum haemorrhage, *MFSI* modified fatigue symptom inventory, *SD* standard deviation, *Dif* difference, *CI* confidence interval, *Hb* haemoglobin, *RBC* red blood cell, *IV* intravenous, *TXA* tranexamic acid

^a^Women who were too ill to do the walk test were coded as 0 m walked and those who did not complete it for other reasons were excluded from the analysis

^b^Postpartum Hb corrected for RBC transfusions and IV fluids received between randomisation and postpartum Hb test

### Clinical diagnosis of PPH

In this population of anaemic women, 7% had a clinical diagnosis of PPH. When compared against shock index ≥1, this outcome measure had 95% specificity, meaning the false positive rate was 5% (see Table 2). Those with a clinical diagnosis of PPH had worse fatigue, reduced ability to exercise and were more breathless after exercise compared to those without this diagnosis (Table 3).

### Estimated blood loss ≥500 ml

Blood loss was estimated to be ≥500 ml in 8% of women. When compared against shock index ≥1, this outcome had 93% specificity, meaning a false positive rate of 7% (see Table 2). Those with blood loss ≥500 ml had worse fatigue, reduced ability to exercise and were more breathless after exercise compared to those with blood loss < 500 ml (see Table 3).

### Proportion of total blood volume lost

**Fig. 1:**
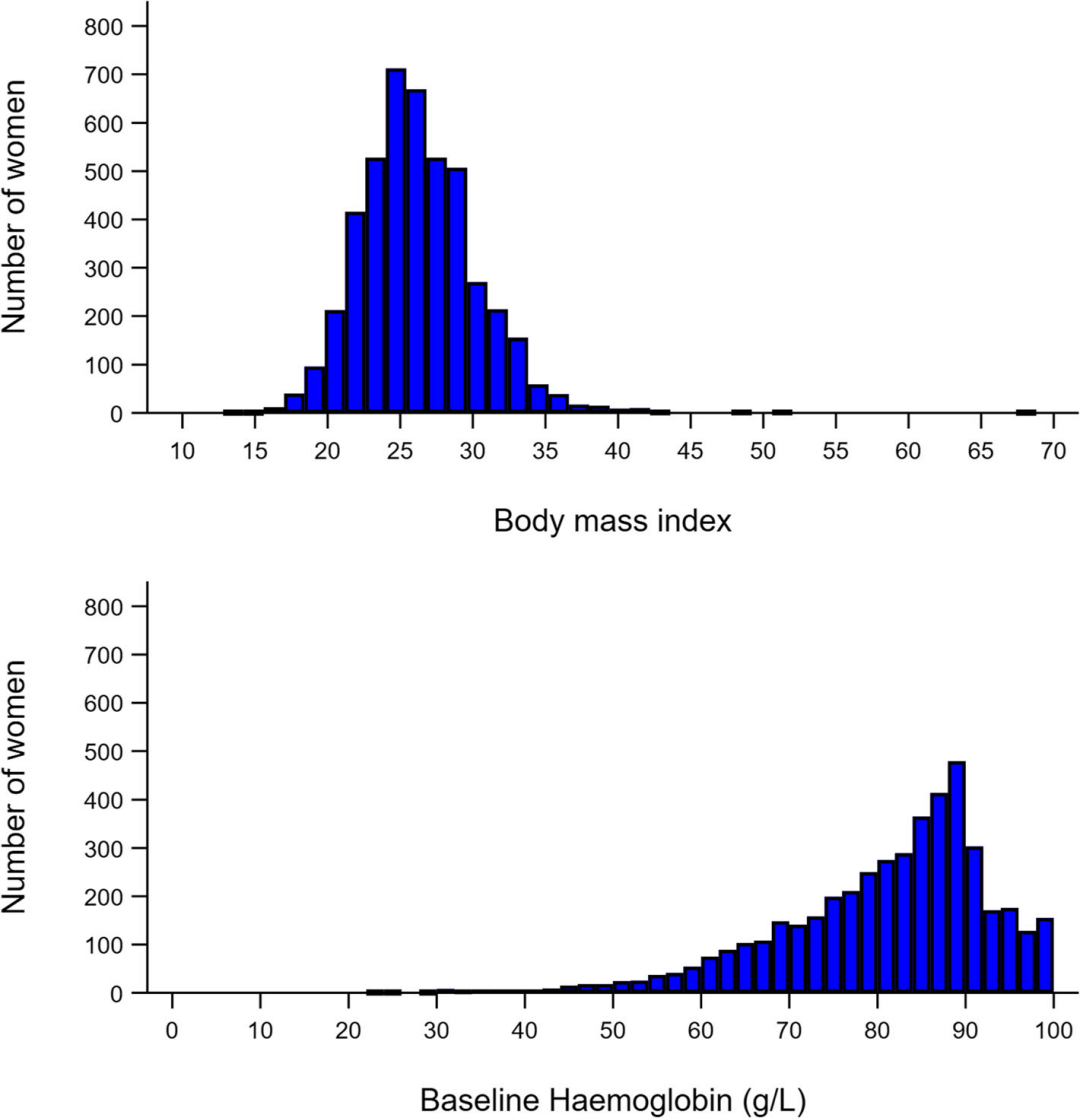
Body mass index and baseline haemoglobin level in the WOMAN-2 trial

Height and weight determine total blood volume. The smaller the woman, the larger the proportion of total blood volume any given volume of blood loss represents. In pregnancy, blood volume per kilogram (kg) decreases with increasing body mass index (BMI) because fat tissue is relatively non-vascular [18]. BMI ranged from 13 to 68 kg/m^2^ in our study population (mean 26, SD 4; see Fig. 1). Based on the Advanced Trauma Life Support classification of hypovolaemic shock, we defined PPH as ≥15% total blood volume lost, which occurred in 3% of women. When compared against shock index ≥1, this outcome had 98% specificity, meaning a false positive rate of 2% (see Table 2). Women who lost ≥15% of their total blood volume had worse fatigue and were more breathless after exercise, with weak evidence of a reduced ability to exercise (see Table 3).

### Peripartum haemoglobin change

Studies suggest that postpartum blood loss ≥500 ml confers a Hb drop ≥20 g/L, although this may vary between women and is affected by red blood cell (RBC) transfusion and intravenous (IV) fluids [19–23]. In the WOMAN-2 trial, 25% (*n* = 1143) and 44% (*n* = 2000) of women received a RBC transfusion or IV fluids (mostly crystalloids) between randomisation and their postpartum Hb test, respectively. In a multivariable linear regression model, one unit of RBC increased peripartum Hb by 7.7 g/L (95% CI 7.0 to 8.3), while 1 L of IV fluids reduced it by 1.5 g/L (95% CI − 2.2 to − 0.8), adjusting for baseline Hb and estimated blood loss. Mean Hb increment per unit of RBC transfused increased with lower baseline Hb (9 vs 6 g/L for Hb of 30 and 99 g/L). To correct postpartum Hb for RBC transfusion, we used coefficients from a predictive model of mean Hb increment derived from 23,194 patients in US hospitals who received one unit of RBC, which adjusted for possible effect modification by baseline Hb, BMI and age [22]. To correct for IV fluids, we applied the model coefficient from the WOMAN-2 data.

After correcting for RBC transfusion and IV fluid, 4% of women had a peripartum Hb drop ≥20 g/L. When compared against shock index ≥1, this outcome had 97% specificity, or a false positive rate of 3% (see Table 2). Because baseline Hb varied (mean = 8.1 g/dL, SD 1.4, range = 2.3–9.9; see Fig. 1), we analysed a relative Hb drop ≥10%, which occurred in 16% of women and had 85% specificity for shock index ≥1 or a 15% false positive rate (see Table 2). Women with a Hb drop ≥20 g/L or ≥ 10% had worse fatigue and breathlessness after exercise, but weak evidence of an increased ability to exercise compared to those with Hb drop < 20 g/L or < 10% (see Table 3).

### Calculated blood loss ≥1000 ml

Another way to define PPH is using calculated blood loss (estimated total blood volume × proportional change in peripartum Hb) [24]. After correcting postpartum Hb for RBC transfusions and fluid resuscitation, 8% of women had calculated blood loss ≥1000 ml. When compared against shock index ≥1, this outcome had 93% specificity, or a 7% false positive rate (see Table 2). Women with calculated blood loss ≥1000 ml had worse fatigue and breathlessness after exercise, but weak evidence of an increased ability to exercise compared to those with calculated blood loss < 1000 ml (see Table 3).

### Interventions for blood loss

Blood transfusion, intravenous fluid, TXA and uterotonics are common interventions for postpartum blood loss but are also routinely given for anaemia, dehydration or PPH prophylaxis. Interventions within 24 h after birth are more likely to be for primary PPH. In total, 25% of women had a blood transfusion within 24 h after giving birth, which had 76% specificity for shock index ≥1 (see Table 2). A total of 44% of women received IV fluid within 24 h after birth, which had 58% specificity for shock index ≥1 (see Table 2). In total, 6% of women received TXA within 24 h after birth, which had 96% specificity for shock index ≥1 (see Table 2). A total of 29% of women received postpartum uterotonics (oxytocin, carbetocin, misoprostol, prostaglandins and/or ergometrine), which had 72% specificity for shock index ≥1 (see Table 2). Women who received a blood transfusion had worse fatigue and a reduced ability to exercise, with weak evidence of increased breathlessness compared to those who did not receive a blood transfusion, whereas women who received IV fluids, TXA or postpartum uterotonics had worse fatigue, reduced ability to exercise and worse breathlessness (see Table 3).

### Shock index ≥1

Although shock index was used as a gold standard measure of the cardiovascular impact of bleeding, we assessed its frequency and importance to women as an outcome measure. Shock index was ≥1 in 8% of women. Those with a shock index ≥1 had worse fatigue and breathlessness after exercise, but some evidence of an increased ability to exercise compared to those with shock index < 1 (Table 3).

## Discussion

To obtain an accurate, precise estimate of the treatment effect, outcome measures should be highly specific and reasonably sensitive. To ensure that evidence of effectiveness translates into real benefit for mothers, the outcome should also be important to women. A clinical diagnosis of PPH, the primary outcome in the WOMAN-2 trial, appears to meet these criteria—it was sufficiently frequent, highly specific for clinical signs of early shock and predictive of maternal functioning after birth. Estimated blood loss and receipt of TXA within 24 h of birth also performed well against our criteria.

High-quality data on over 4500 anaemic pregnant women provided reliable estimates of PPH and its association with various factors. We were able to assess several PPH definitions and discern the sequence of events. Blood loss was estimated visually rather than measured as it is more practical and no worse at predicting adverse maternal outcomes [25]. The formula to estimate total blood volume was derived from pregnant women (blood volume = weight (kg) × 95 if BMI < 30, or 73 if BMI ≥30) but we did not collect data on pre-pregnancy weight [18]. Hb was measured with the Haemocue Hb 201 system which has reasonable accuracy [26]. We corrected postpartum Hb for RBC transfusion and IV fluid but not for time to postpartum Hb test, which had only a small effect (0.03 g/L drop in postpartum Hb for 1 h increase in time from childbirth to Hb test) [22, 23]. Although unlikely, women could possibly receive a RBC transfusion between their baseline Hb test and randomisation, which is not recorded in the trial. While heart rate and blood pressure can be accurately measured, shock index is an imperfect physiological marker of postpartum blood loss with low sensitivity for PPH [27]. Maternal cardiovascular compensatory mechanisms like haemoconcentration and increased cardiac output after childbirth may obscure early physiologic signs of postpartum bleeding. Shock can be caused by other conditions like sepsis, although this affected < 1% of trial participants.

By combining clinical judgement, physical signs of haemodynamic instability and estimated blood loss, a clinical diagnosis of PPH may be more specific for significant bleeding than estimated blood loss alone, particularly in anaemic women [28]. The TRAAP trial of TXA for the prevention of blood loss after vaginal birth found a 17% reduction in blood loss ≥500 ml with TXA (RR = 0.83, 95% CI 0.68–1.01) but a 26% reduction in clinically diagnosed PPH (RR = 0.74, 95% CI 0.61–0.91) [29]. Calculated blood loss combines peripartum Hb change and total blood volume. The TRAAP2 trial of TXA for PPH prevention in Caesarean births found a reduction in calculated blood loss ≥1000 ml or transfusion (RR = 0.84, 95% CI 0.75–0.94) [24]. However, surrogate measures of PPH based on Hb change may lack value to patients and clinical relevance. Of note, we found a non-significant increase in ability to exercise among women with PPH defined using peripartum Hb change. The relationship between Hb level and postpartum blood loss is not straightforward [20]. Dehydration during childbirth can cause haemoconcentration, increasing postpartum Hb [30]. Physiological adaptions of pregnancy like increased plasma volume and haemodilution may prevent a large drop in Hb with postpartum bleeding [20, 31]. Indeed, few women in the WOMAN-2 trial experienced a Hb drop ≥20 g/L.

Blood transfusion, IV fluid and uterotonics had low specificity, probably because some were given routinely for reasons other than bleeding or despite blood loss. The WOMAN trial of TXA for PPH showed that early treatment reduces death due to bleeding (RR = 0·69, 95% CI 0·52–0·91) but there was no effect on all-cause mortality or hysterectomy as TXA cannot influence non-bleeding causes of death (29% of all deaths) or hysterectomies planned before randomisation (38% of hysterectomies for bleeding occurred within an hour) [12, 32]. Careful consideration of the mechanism of action of the trial treatment, the natural history of the disease and potential sources of null bias is vital when selecting primary outcomes for clinical trials.

The WOMAN-2 trial will provide further insight into outcome measures for PPH research in anaemic women and evidence on the role of TXA for PPH prevention. Anaemia is a highly prevalent risk factor for PPH which needs more attention if we are to reduce the burden of PPH and its consequences for anaemic women and their babies [3, 6]. Large high-quality randomised trials are needed to find effective interventions for the treatment of anaemia in women of reproductive age.

## Conclusions

Outcome measures in clinical trials of interventions for PPH prevention should facilitate valid and precise estimation of the treatment effect and be important to women. A clinical diagnosis of PPH is highly specific for the cardiovascular effects of significant postpartum bleeding, sufficiently common and associated with maternal functioning after birth, making it an appropriate primary outcome for the WOMAN-2 trial.

## Data Availability

The datasets generated and/or analysed during the current study are not publicly available because the WOMAN-2 trial is ongoing. After trial completion and publication of the planned primary and secondary analyses, the dataset will be made publicly available on the Free Bank of Injury and Emergency Research Data (freeBIRD) website, our data-sharing portal at https://ctu-app.lshtm.ac.uk/freebird/.

## Abbreviations

CI: Confidence interval
Hb: Haemoglobin
MSFI: Modified fatigue symptom inventory
PPH: Postpartum haemorrhage
RR: Relative risk/risk ratio
TXA: Tranexamic acid
WHO: World Health Organization
WOMAN-2 trial: WOrld Maternal ANtifibrinolytic-2 trial

## References

[1] Say L, Chou D, Gemmill A, Tunçalp Ö, Moller AB, Daniels J, Gülmezoglu AM, Temmerman M, Alkema L (2014). Global causes of maternal death: a WHO systematic analysis. Lancet Glob Health.

[2] Nair M, Choudhury MK, Choudhury SS, Kakoty SD, Sarma UC, Webster P (2016). Association between maternal anaemia and pregnancy outcomes: a cohort study in Assam, India. BMJ Glob Health.

[3] Parks S, Hoffman MK, Goudar SS, Patel A, Saleem S, Ali SA, Goldenberg RL, Hibberd PL, Moore J, Wallace D, McClure E, Derman RJ (2019). Maternal anaemia and maternal, fetal, and neonatal outcomes in a prospective cohort study in India and Pakistan. BJOG An Int J Obstet Gynaecol.

[4] Stevens GA, Finucane MM, De-Regil LM, Paciorek CJ, Flaxman SR, Branca F (2013). Global, regional, and national trends in haemoglobin concentration and prevalence of total and severe anaemia in children and pregnant and non-pregnant women for 1995-2011: a systematic analysis of population-representative data. Lancet Glob Health.

[5] Daru MBBSJ, Zamora J, Thangaratinam S, Khan KS, Epidemiology C, Health P (2018). Risk of maternal mortality in women with severe anaemia during pregnancy and post partum: a multilevel analysis. Lancet Glob Health.

[6] Guignard J, Deneux-Tharaux C, Seco A, Beucher G, Kayem G, Bonnet MP, Langer B, Dupont C, Rudigoz RC, Venditelli F, Rozenberg P, Carbillon L, Azria E, Baunot N, Crenn-Hebert C, Fresson J, Mignon A, Bouvier-Colle MH, Chantry A, Chiesa-Dubruille C, the EPIMOMS group (2021). Gestational anaemia and severe acute maternal morbidity: a population-based study. Anaesthesia..

[7] World Health Organization (2017). WHO recommendations for the prevention and treatment of postpartum haemorrhage.

[8] World Health Organization (WHO) (1989). The Prevention and Management of Postpartum Haemorrhage.

[9] Meher S, Cuthbert A, Kirkham J, Williamson P, Abalos E, Aflaifel N, et al. Core outcome sets for prevention and treatment of postpartum haemorrhage: an international Delphi consensus study. BJOG An Int J Obstet Gynaecol. 2018; Available from: http://doi.wiley.com/10.1111/1471-0528.15335. [cited 2018 Aug 23]. 10.1111/1471-0528.15335 29920912

[10] Ker K, Roberts I, Chaudhri R, Fawole B, Beaumont D, Balogun E (2018). Tranexamic acid for the prevention of postpartum bleeding in women with anaemia: study protocol for an international, randomised, double-blind, placebo-controlled trial. Trials.

[11] ICH (1998). Harmonised Tripartite Guideline E9 Statistical Principles for Clinical Trials.

[12] Brenner A, Arribas M, Cuzick J, Jairath V, Stanworth S, Ker K (2018). Outcome measures in clinical trials of treatments for acute severe haemorrhage. Trials.

[13] Copeland KT, Checkoway H, Mcmichael AJ, Holbrook RH (1977). Bias due to misclassification in the estimation of relative risk. Am J Epidemiol.

[14] Pacagnella RC, Souza JP, Durocher J, Perel P, Blum J, Winikoff B, et al. A systematic review of the relationship between blood loss and clinical signs. PLoS One. 2013;8 Available from: https://pubmed.ncbi.nlm.nih.gov/23483915/. [cited 2020 Aug 6]. 10.1371/journal.pone.0057594 PMC3590203 23483915

[15] El Ayadi AM, Nathan HL, Seed PT, Butrick EA, Hezelgrave NL, Shennan AH, et al. Vital sign prediction of adverse maternal outcomes in women with hypovolemic shock: the role of shock index. Raju R, editor. PLoS One. 2016;11(2):e0148729. Available from: https://dx.plos.org/10.1371/journal.pone.0148729. [cited 2020 Aug 6] 10.1371/journal.pone.0148729 PMC4762936 26901161

[16] Nathan HL, El Ayadi A, Hezelgrave NL, Seed P, Butrick E, Miller S (2015). Shock index: an effective predictor of outcome in postpartum haemorrhage?. BJOG An Int J Obstet Gynaecol.

[17] Le Bas A, Chandraharan E, Addei A, Arulkumaran S (2014). Use of the “obstetric shock index” as an adjunct in identifying significant blood loss in patients with massive postpartum hemorrhage. Int J Gynecol Obstet.

[18] Vricella LK, Louis JM, Chien E, Mercer BM. Blood volume determination in obese and normal-weight gravidas: the hydroxyethyl starch method. Am J Obstet Gynecol Mosby Inc. 2015:408.e1–6 Available from: /pmc/articles/PMC4589161/?report = abstract. [cited 2020 Aug 13]. 10.1016/j.ajog.2015.05.021 PMC4589161 25981844

[19] Dupont C, Touzet S, Colin C, Deneux-Tharaux C, Rabilloud M, Clement HJ, Lansac J, Colle MHB, Rudigoz RC (2009). Incidence and management of postpartum haemorrhage following the dissemination of guidelines in a network of 16 maternity units in France. Int J Obstet Anesth.

[20] Anger H, Durocher J, Dabash R, Winikoff B. How well do postpartum blood loss and common definitions of postpartum hemorrhage correlate with postpartum anemia and fall in hemoglobin? PLoS One. 2019;14(8) Available from: /pmc/articles/PMC6705817/?report = abstract. [cited 2020 Aug 13]. 10.1371/journal.pone.0221216 PMC6705817 31437195

[21] Yefet E, Yossef A, Suleiman A, Hatokay A, Nachum Z (2020). Hemoglobin drop following postpartum hemorrhage. Sci Rep.

[22] Roubinian NH, Plimier C, Woo JP, Lee C, Bruhn R, Liu VX (2019). Effect of donor, component, and recipient characteristics on hemoglobin increments following red blood cell transfusion. Blood.

[23] Perel A (2017). Iatrogenic hemodilution: a possible cause for avoidable blood transfusions?. Crit Care BioMed Central Ltd.

[24] Sentilhes L, Senat M-V, Le Lous M, Winer N, Rozenberg P, Kayem G (2021). Tranexamic acid for the prevention of postpartum hemorrhage after cesarean delivery: the TRAAP2 trial. Am J Obstet Gynecol.

[25] Diaz V, Abalos E, Carroli G. Methods for blood loss estimation after vaginal birth. Cochrane Database Syst Rev John Wiley and Sons Ltd. 2018;2018 Available from: /pmc/articles/PMC6513177/?report = abstract. [cited 2020 Sep 30]. 10.1002/14651858.CD010980.pub2 PMC6513177 30211952

[26] Hiscock R, Kumar D, Simmons SW (2015). Systematic review and meta-analysis of method comparison studies of Masimo pulse co-oximeters (Radical-7^TM^ or Pronto-7^TM^) and HemoCue® absorption spectrometers (B-Hemoglobin or 201+) with laboratory haemoglobin estimation. Anaesth Intensive Care.

[27] Ushida T, Kotani T, Imai K, Nakano-Kobayashi T, Nakamura N, Moriyama Y (2021). Shock index and postpartum hemorrhage in vaginal deliveries: a multicenter retrospective study. Shock.

[28] Borovac-Pinheiro A, Pacagnella RC, Cecatti JG, Miller S, El Ayadi AM, Souza JP (2018). Postpartum hemorrhage: new insights for definition and diagnosis. Am J Obstet Gynecol.

[29] Sentilhes L, Winer N, Azria E, Sénat M-V, Le Ray C, Vardon D (2018). Tranexamic acid for the prevention of blood loss after vaginal delivery. N Engl J Med.

[30] Watanabe T, Minakami H, Sakata Y, Matsubara S, Tamura N, Obara H (2001). Effect of labor on maternal dehydration, starvation, coagulation, and fibrinolysis. J Perinat Med.

[31] Pritchard JA (1965). Changes in the blood volume during pregnancy and delivery. Anesthesiology.

[32] WOMAN Trial Collaborators (2017). Effect of early tranexamic acid administration on mortality, hysterectomy, and other morbidities in women with post-partum haemorrhage (WOMAN): an international, randomised, double-blind, placebo-controlled trial. Lancet.

